# Strengthening research capacity through regional partners: the HRP Alliance at the World Health Organization

**DOI:** 10.1186/s12978-020-00965-0

**Published:** 2020-08-26

**Authors:** Richard Adanu, Luis Bahamondes, Vanessa Brizuela, Evelyn Gitau, Seni Kouanda, Pisake Lumbiganon, Thi Thuy Hanh Nguyen, Sarah Saleem, Anna Thorson, Kwasi Torpey

**Affiliations:** 1 Department of Population, Family and Reproductive Health, University of Ghana School of Public Health, Accra, Ghana; 2 Department of Obstetrics and Gynaecology, University of Campinas Medical Faculty, Campinas, SP Brazil; 3 UNDP/UNFPA/UNICEF/WHO/World Bank Special Programme of Research, Development and Research Training in Human Reproduction (HRP), Department of Sexual and Reproductive Health and Research, World Health Organization, Avenue Appia 20, 1211 Geneva, Switzerland; 4 African Population and Health Research Center, Nairobi, Kenya; 5 Institut de Recherche en Sciences de la Santé (IRSS), Ouagadougou, Burkina Faso; 6 Department of Obstetrics and Gynaecology, Faculty of Medicine, Khon Kaen University, Khon Kaen, Thailand; 7 Institute for Preventive Medicine and Public Health, Hanoi Medical University, Hanoi, Viet Nam; 8 Department of Community Health Sciences, Aga Khan University, Karachi, Pakistan

**Keywords:** Research capacity strengthening, Research, Sexual and reproductive health, LMIC, Renforcement des capacités de recherche, Recherche, Santé sexuel et reproductif, PFR-PRI, Reforço da capacidade de pesquisa, Pesquisa, Saúde sexual e reprodutiva, Países de baixa e média renda, Fortalecimiento de la capacidad de Investigación, Investigación, Salud sexual y reproductiva, Países de ingresos bajos y medianos

## Abstract

**Background:**

Improvements in health cannot occur without cutting-edge research informing the design and implementation of health programmes and policies, highlighting the need for qualified and capable researchers and institutions in countries where disease burden is high and resources are limited.

**Main body:**

Research capacity strengthening efforts in low- and middle-income countries have included provision of training scholarships for postgraduate degrees, often in high-income countries, internships at research universities/centres, short courses, as well as involvement with research groups for hands-on experience, among others. The HRP Alliance provides opportunities for developing local research capacity in sexual and reproductive health and rights through institutions based in low- and middle-income countries linked with ongoing and past collaborative studies. It is a network of HRP research partner institutions, World Health Organization (WHO) country and regional offices, WHO special programmes and partnerships, and WHO collaborating centres.

**Conclusion:**

It is through the HRP Alliance that HRP seeks to improve population health by strengthening local research capacity in sexual and reproductive health across the globe, with focus in low- and middle-income countries, in alignment with WHO’s quest of promoting healthier populations.

## Résumé

**Introduction:**

Il ne peut y avoir d’amélioration en matière de santé sans une recherche de pointe qui éclaire la conception et la mise en oeuvre des programmes et des politiques de santé, en soulignant la nécessité de disposer de chercheurs et d’institutions qualifiés et compétents dans les pays où la charge de morbidité est élevée et où les ressources sont limitées.

**Texte principal:**

Parmi les efforts de renforcement des capacités de recherche dans les pays à faible revenu et pays à revenu intermédiaire (PFR-PRI), figurent l’octroi de bourses de formation pour des diplômes de troisième cycle, souvent dans des pays à revenu élevé, des stages dans des universités/centres de recherche, des cours de courte durée, ainsi que la participation à des groupes de recherche pour une expérience pratique, entre autres. L’Alliance HRP offre des possibilités de développement des capacités de recherche locales en matière de santé et des droits sexuels et reproductifs (SDSR), par l’intermédiaire d’institutions basées dans les pays à revenu faible et intermédiaire, en lien avec les études collaboratives en cours et passées. Il s’agit d’un réseau d’institutions partenaires de recherche du HRP, de bureaux nationaux et régionaux de l’OMS, de programmes spéciaux et de partenariats de l’OMS et de centres collaborateurs de l’OMS.

**Conclusion:**

C’est par l’intermédiaire de l’Alliance HRP que le HRP cherche à améliorer la santé des populations en renforçant les capacités de recherche locales en matière de SDSR dans le monde entier, en particulier dans les PRF-PRI, conformément à la mission de l’OMS de promouvoir la bonne santé des populations.

## Resumo

**Introducção:**

As melhorias na saúde não podem ocorrer sem as pesquisas de ponta que informam o planejamento e a implementação de programas e políticas de saúde, evidenciando a necessidade de pesquisadores e instituições capazes e qualificadas em países onde a incidência de doença é alta, e os recursos são limitados.

**Texto principal:**

O empenho pelo reforço da capacidade de pesquisa em países de baixa e média renda (LMIC) tem incluído provisões de bolsas de formação para pós-graduação, geralmente em países de renda alta, estágios em universidades/centros de pesquisas, cursos de curta duração, assim como envolvimento com grupos de pesquisas para experiência prática, entre outros. A HRP Alliance fornece oportunidades para o desenvolvimento de capacidade de pesquisa local em saúde e direitos sexuais e reprodutivos (SRHR) através de instituições estabelecidas em países de baixa e média renda ligados a estudos colaborativos anteriores e em andamento. É uma rede de instituições parceiras HRP, escritórios nacionais e regionais da OMS, programas e parcerias especiais e centros colaborativos da OMS.

**Conclusão:**

É através da HRP Alliance que o desenvolvimento e treinamento em pesquisa em reprodução humana (HRP) busca melhorar a saúde da população pelo reforço local na capacidade de pesquisa em SRHR em todo o mundo, com foco nos LMIC, em alinhamento com a busca da OMS em promover populações mais saudáveis.

## Resumen

**Introducción:**

Las mejoras en la salud no pueden suceder sin investigaciones de vanguardia que informen sobre el diseño y la implementación de programas y políticas de salud y que pongan énfasis en la necesidad de contar con investigadores e instituciones capaces y competentes en los países donde la carga de morbilidad es alta y los recursos son limitados.

**Texto principal:**

Los esfuerzos para el fortalecimiento de la capacidad de investigación en los países de ingresos bajos y medianos incluyeron, entre otros, el ofrecimiento de becas de capacitación a graduados universitarios, a menudo en países de ingresos altos, pasantías en universidades y centros de investigación, cursos breves y también participación en grupos de investigación para experiencias prácticas. La Alianza HRP ofrece oportunidades para el desarrollo de capacidades de investigación locales en salud y derechos sexuales y reproductivos (SDSR) a través de instituciones ubicadas en países de ingresos bajos y medianos vinculadas con estudios colaborativos presentes y pasados. Se trata de una red de instituciones asociadas de investigación del Programa de Reproducción Humana (HRP), oficinas regionales y nacionales de la OMS, programas y asociaciones especiales de la OMS y centros colaboradores de la OMS.

**Conclusión:**

A través de la Alianza HRP, el HRP busca mejorar la salud de la población con el fortalecimiento de la capacidad de investigación local en SDSR en todo el mundo, centrado en los países de ingresos bajos y medianos, y alineado con la misión de la OMS de promover poblaciones más saludables.

## Background

Improvements in health cannot occur without cutting-edge research informing the design and implementation of health programmes and policies. Evidence generated in-country is necessary to shape and add relevance to national research and policy agendas [1–3]. This highlights the need for qualified and capable researchers and institutions in countries where disease burden is high and resources are limited. The inverse relationship between sexual and reproductive health and rights (SRHR)-related disease burden and availability of research capacity is recognised and different efforts have been undertaken to strengthen capacity in low-and-middle-income countries (LMIC) over the last few decades [4].

## Main body

Research capacity strengthening (RCS) efforts in LMICs have included provision of training scholarships for postgraduate degrees, often in high-income countries, internships at research universities/centres, short courses, as well as involvement with research groups for hands-on experience, among others [5–7]. These efforts have resulted in producing researchers for LMICs but have not always significantly built the capacity of LMIC institutions to conduct their own research to identify solutions or monitor uptake and use of evidence for better SRHR outcomes [6]. Sustainable RCS should develop the capacity of target institutions in countries in need so they can successfully train competent researchers, and respond to local, regional, and global agendas [5, 6]. While currently there are well-established research institutes in LMICs that can train and conduct high quality research, there are still gaps with regards to strengthening the research capacity of junior researchers. Ensuring equitable and fair authorship is also critical to developing local RCS [8–10].

### The HRP Alliance

The HRP Alliance, created in 2016 as part of the UNDP/UNFPA/UNICEF/WHO/World Bank Special Programme of Research, Development and Research Training in Human Reproduction (HRP) [11], provides opportunities for developing local research capacity through institutions linked with ongoing and past collaborative studies (visit website using this link https://www.who.int/reproductivehealth/hrp_alliance/en/). It is a network of HRP research partner institutions, WHO country and regional offices, WHO special programmes and partnerships, and WHO collaborating centres. It is through the HRP Alliance that HRP seeks to improve population health by strengthening local research capacity in SRHR across the globe, with focus in LMICs, in alignment with WHO’s quest of promoting healthier populations [12]. (See Table 1). Despite the recent creation of the HRP Alliance, HRP has been leading RCS efforts for several decades. In the past, this has been done through long-term institutional development grants which focused mostly on individual institutional strengthening and support of local research projects. The focus now, through the HRP Alliance, lies on building a regional critical mass of researchers supported by institutions located in the regions, illustrating a true horizontal collaboration among researchers.

**Table 1:** The HRP Alliance – vision, mission, strategy, goals and core values

**Vision** The HRP Alliance aims to improve sexual and reproductive health and rights (SRHR) globally by strengthening research capacity. **Mission** To support institutions to develop high quality research capacity in SRHR. **Strategy** By linking research capacity strengthening (RCS) with HRP research and knowledge transfer, the HRP Alliance capitalizes and reinforces existing collaborations. It provides support for institutions to position themselves in the global SRHR research and knowledge transfer arena. This is provided through long-term institutional grants to research institutions selected as regional RCS hubs supporting other institutions in their respective regions. **Goals** 1- To strengthen SRHR research capacity in an alliance of institutions and stakeholders in LMICs 2- To improve institutions’ own research infrastructure 3- To strengthen research capacity of the institutions in the region through trainings, courses, and formal education of individuals 4- To link HRP research with HRP Alliance partners on SRHR topics 5- To lead in knowledge translation activities 6- To build a critical mass of world class researchers in SRHR implementation research around the globe 7- To support research in humanitarian or emergency SRHR issues **Core values** • Focus on gender equality • Promote rights-based research • Lead in high level implementation research • Foster knowledge translation among a global network of SRHR researchers **Website**: https://www.who.int/reproductivehealth/hrp_alliance/en/	

**Fig. 1:**
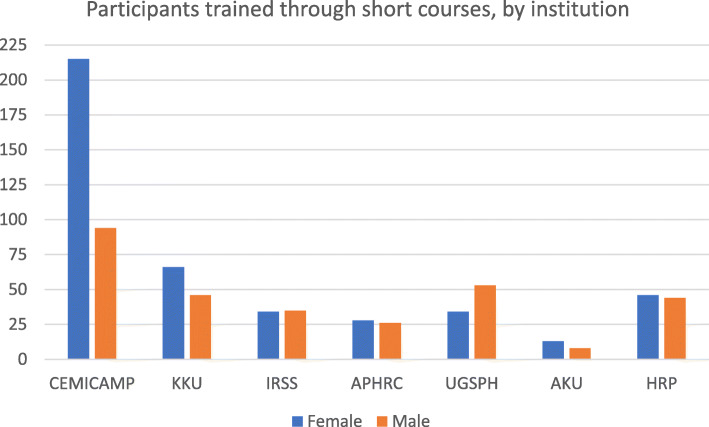
Individuals trained through courses offered by HRP Alliance hubs or HRP Alliance in headquarters

**Fig. 2:**
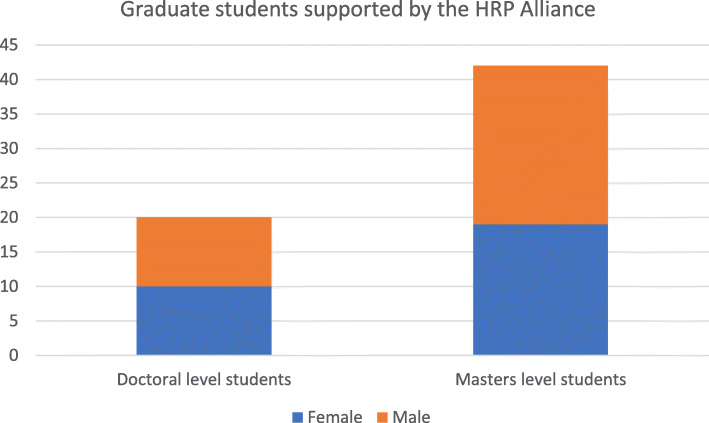
Doctoral and master’s students receiving scholarships through the HRP Alliance to complete their studies

At the core of the HRP Alliance are regional RCS “hubs,” selected through an open competitive process that considers experience in SRHR RCS and capacity to provide regional leadership in RCS. These hubs, based in Brazil, Burkina Faso, Ghana, Kenya, Pakistan, Thailand, and Viet Nam,^1^ are entrusted with providing RCS support to institutions in their regions. While the HRP Alliance is still in its nascent stages, support is provided primarily through:

- Workshops and trainings on SRHR, research methodologies and biostatistics, systematic review and meta-analysis, qualitative research methods, implementation research, monitoring and evaluation, protocol development and manuscript writing (Fig. 1);
- Post-graduate education specific to SRHR research (through masters and/or doctoral degrees) (Fig. 2);
- Tailored support to country research institutions in the development and implementation of research studies and the production of scientific publications;
- Leadership in knowledge transfer activities that contribute to ensuring the implementation of WHO recommendations for policy and practice;
- Collaborative grant proposals among several hubs or institutions supported by the hubs using the HRP Alliance network to leverage experience and expertise;
- Enabled collaborations among HRP Alliance fellows for specific research projects;
- Response to health emergencies through SRHR research to improve rapid health system response and local research capacity.

Since inception, the HRP Alliance has trained over 700 participants from LMICs through 30 workshops and courses and is supporting over 60 researchers obtain a master’s or doctoral degree, some of whom have been involved in the local implementation of HRP multi-country studies [13–17] and in secondary analyses. The HRP Alliance supports the use of group authorship for multi-country studies, local leadership in secondary and country-specific analyses, and the establishment of authorship rules and roles prior to project start-up. Over 20 research groups from Latin America have been funded by the HRP Alliance to provide the evidence base to respond to the Zika virus epidemic in 2016–2017 [18] and the mass migration crisis in the Americas in 2019–2020. The HRP Alliance also swiftly responds to the needs arising from health and humanitarian emergencies according to the specific needs for RCS and research. In the upcoming years, the HRP Alliance will support junior researchers through a tailored mentorship programme for women and post-doctoral fellowships as well as support additional research to study the SRHR of migrants in the Eastern Mediterranean region.

## Conclusion

The HRP Alliance model for RCS is one among many, but it holds the unique characteristic of allowing for the development and building of research capacity of individuals and institutions through the engagement and leadership of research institutions located in LMICs. This model has the potential, by supporting RCS activities through institutions located in the regions of interest, to prevent future brain drain of qualified researchers by building capacity and offering viable opportunities for implementing research in their home countries. This paper serves as a blueprint of what the HRP Alliance has set out to do and to be held accountable to its mandate.

## Supplementary information

**Additional file 1.** Version de l'article en français - versão do artigo em português - versión del artículo en español

## Data Availability

Not applicable.

## Abbreviations

*HRP*: Human Reproduction Programme, the UNDP/UNFPA/UNICEF/WHO/World Bank Special Programme of Research, Development and Research Training in Human Reproduction
*LMIC*: low- and middle-income countries
*RCS*: research capacity strengthening
*SRHR*: sexual and reproductive health and rights
*WHO*: World Health Organization

## Footnotes

1 Brazil: Centro de Pesquisas em Saúde Reprodutiva de Campinas – CEMICAMP; Burkina Faso: Institut de Recherche en Sciences de la Santé – IRSS; Ghana: University of Ghana School of Public Health – UGSPH; Kenya: African Population Health Research Center – APHRC; Pakistan: Aga Khan University – AKU; Thailand: Khon Kaen University – KKU; and Viet Nam: Hanoi Medical University – HMU.
