# Supplementary material for: Strengthening research capacity through regional partners: the HRP Alliance at the World Health Organization
Source: Reprod Health. 2020 Aug 26;17:131. doi: 10.1186/s12978-020-00965-0 (PMC7448306; doi:10.1186/s12978-020-00965-0)
Supplement: Supplementary file 1 — Additional file 1. Version de l'article en français - versão do artigo em português - versión del artículo en español [file 12978_2020_965_MOESM1_ESM.zip › HRP RCS commentary_FR.pdf]

Renforcer les capacités de recherche par le biais de partenaires régionaux : l'Alliance HRP à l'Organisation mondiale de la santé

Résumé

Introduction : Il ne peut y avoir d'amélioration en matière de santé sans une recherche de pointe qui éclaire la conception et la mise en œuvre des programmes et des politiques de santé, en soulignant la nécessité de disposer de chercheurs et d'institutions qualifiés et compétents dans les pays où la charge de morbidité est élevée et où les ressources sont limitées.

Texte principal : Parmi les efforts de renforcement des capacités de recherche dans les pays à faible revenu et pays à revenu intermédiaire (PFR-PRI), figurent l'octroi de bourses de formation pour des diplômes de troisième cycle, souvent dans des pays à revenu élevé, des stages dans des universités/centres de recherche, des cours de courte durée, ainsi que la participation à des groupes de recherche pour une expérience pratique, entre autres. L'Alliance HRP offre des possibilités de développement des capacités de recherche locales en matière de santé et des droits sexuels et reproductifs, par l'intermédiaire d'institutions basées dans les pays à revenu faible et intermédiaire, en lien avec les études collaboratives en cours et passées. Il s'agit d'un réseau d'institutions partenaires de recherche du HRP, de bureaux nationaux et régionaux de l'OMS, de programmes spéciaux et de partenariats de l'OMS et de centres collaborateurs de l'OMS.

Conclusion : C'est par l'intermédiaire de l'Alliance HRP que le HRP cherche à améliorer la santé des populations en renforçant les capacités de recherche locales en matière de SDSR dans le monde entier, en particulier dans les PRF-PRI, conformément à la mission de l'OMS de promouvoir la bonne santé des populations.

Mots clés : renforcement des capacités de recherche, recherche, santé sexuel et reproductif, PFR-PRI

Introduction

Les améliorations en termes de santé ne peuvent se produire sans une recherche de pointe qui éclaire la conception et la mise en œuvre des programmes et des politiques de santé. Les éléments probants générés dans le pays sont nécessaires pour façonner et ajouter de la pertinence aux programmes nationaux de recherche et de politique.(1–3) Cela souligne la nécessité de disposer de chercheurs et d'institutions qualifiés et compétents dans les pays où la charge de morbidité est élevée et où les ressources sont limitées. La relation inverse entre la charge de morbidité liée à la santé et aux droits sexuels et reproductifs (SDSR) et la disponibilité des capacités de recherche est reconnue et différents efforts ont été entrepris pour renforcer les capacités dans les pays à revenu faible et intermédiaire (PRF-PRI) au cours des dernières décennies.(4)

Texte principal

Parmi les efforts de renforcement des capacités de recherche dans les PFR-PRI, figurent l'octroi de bourses de formation pour des diplômes de troisième cycle, souvent dans des pays à revenu élevé, des stages dans des universités/centres de recherche, des cours de courte durée, ainsi que la participation à des groupes de recherche pour une expérience pratique, entre autres.(5–7) Ces efforts ont permis de recruter des chercheurs pour les PFR-PRI, mais n'ont pas toujours renforcé de manière significative la capacité de leurs institutions à mener leurs propres recherches pour identifier des solutions ou superviser leur adoption et leur utilisation, afin d'obtenir de meilleurs résultats en matière de SDSR.(6) Le renforcement des capacités de recherche durable doit développer la capacité des institutions cibles dans les pays qui en ont besoin, afin qu'elles puissent former avec succès des chercheurs compétents, et répondre aux priorités locales, régionales et mondiales.(5,6) Bien qu'il existe actuellement des instituts de recherche bien établis dans les PFR-PRI qui peuvent former et mener des recherches de haute qualité, il existe encore des lacunes en ce qui concerne le renforcement de la capacité de recherche des jeunes chercheurs. La garantie d'une paternité équitable et juste est également essentielle au développement du renforcement des capacités de recherche durable au niveau local.(8–10)

L'Alliance HRP

L'Alliance HRP, créée en 2016 dans le cadre du Programme spécial PNUD/FNUAP/UNICEF/OMS/Banque mondiale de recherche, de développement et de formation à la recherche en reproduction humaine (HRP),(11) offre des possibilités de développement des capacités de recherche locales par le biais d'institutions liées à des études collaboratives en cours et antérieures (visitez le site web en cliquant sur ce lien). Il s'agit d'un réseau d'institutions partenaires de recherche du HRP, de bureaux nationaux et régionaux de l'OMS, de programmes spéciaux et de partenariats de l'OMS et de centres collaborateurs de l'OMS. C'est par l'intermédiaire de l'Alliance HRP que le HRP cherche à améliorer la santé des populations en renforçant les capacités de recherche locales en matière de SDSR dans le monde entier, en particulier dans les PRF-PRI, conformément à la mission de l'OMS de promouvoir la bonne santé des populations.(12) (Voir le panneau). Malgré la récente création de l'Alliance HRP, HRP dirige les efforts de renforcer les capacités de recherche depuis plusieurs décennies. Dans le passé, cela a été fait grâce à des subventions de développement institutionnel à long terme qui se sont principalement concentrées sur le renforcement institutionnel individuel et le soutien de projets de recherche locaux. Aujourd'hui, à travers l'Alliance HRP, l'accent est mis sur la constitution d'une masse critique régionale de rechercheurs soutenus par des institutions situées dans les régions, illustrant une véritable collaboration horizontale entre les rechercheurs.

Au cœur de l'Alliance HRP se trouvent les « centres » de renforcement des capacités de recherche régionaux, sélectionnés par un processus concurrentiel ouvert qui tient compte de l'expérience en matière de renforcement des capacités de recherche en termes de SDSR et de la capacité à assurer un

leadership régional en matière de renforcement des capacités de recherche. Ces centres, basés au Brésil, au Burkina Faso, au Ghana, au Kenya, au Pakistan, en Thaïlande et au Vietnam,* sont chargés de fournir un soutien au niveau du renforcement des capacités de recherche aux institutions de leur région. Bien que l'Alliance HRP n'en soit qu'à ses débuts, l'aide est principalement fournie par le biais de :

- Ateliers et formations sur les SDSR, les méthodologies de recherche et les biostatistiques, l'examen systématique et la méta-analyse, les méthodes de recherche qualitative, la recherche de mise en œuvre, le suivi et l'évaluation, l'élaboration de protocoles et la rédaction de manuscrits (organigramme 1) ;
- Enseignement postuniversitaire spécifique à la recherche SDSR (par le biais de masters et/ou de doctorats) (organigramme 2) ;
- Soutien personnalisé aux institutions de recherche des pays dans l'élaboration et la mise en œuvre d'études de recherche et la production de publications scientifiques ;
- Leadership dans les activités de transfert de connaissances qui contribuent à assurer la mise en œuvre des recommandations de l'OMS en matière de politique et de pratique ;
- Propositions de subventions collaboratives parmi plusieurs centres ou institutions soutenus par les centres utilisant le réseau de l'Alliance HRP pour tirer parti de l'expérience et de l'expertise ;
- Collaboration permise entre les membres de l'Alliance HRP pour des projets de recherche spécifiques ;
- Réponse aux urgences sanitaires par la recherche en matière de SDSR afin d'améliorer la rapidité de réaction du système de santé et la capacité de recherche locale.

Depuis sa création, l'Alliance HRP a formé plus de 700 participants issus des PRF-PRI dans le cadre de 30 ateliers et cours et soutiennent plus de 60 chercheurs dans l'obtention d'une maîtrise ou d'un doctorat, dont certains ont participé à la mise en œuvre locale des études multinationales du HRP(13–17) et à des analyses secondaires. L'Alliance HRP soutient l'utilisation de la paternité collective pour les études multinationales, le leadership local dans les analyses secondaires et spécifiques à un pays, et l'établissement de règles et de rôles pour les auteurs avant le démarrage du projet. Plus de 20 groupes de recherche d'Amérique latine ont été financés par l'Alliance HRP pour fournir la base d'éléments probants permettant de répondre à l'épidémie du virus Zika en 2016-2017 (18) et à la crise des migrations massives dans les Amériques en 2019-2020. L'Alliance HRP répond également rapidement aux besoins découlant des urgences sanitaires et humanitaires en fonction des besoins spécifiques en matière de recherche et de renforcement des capacités de recherche. Dans les années à venir, l'Alliance HRP soutiendra les jeunes chercheurs grâce à un programme de mentorat adapté aux femmes et à des bourses postdoctorales, ainsi qu'à des recherches supplémentaires pour étudier les SDSR des migrants dans la région de la Méditerranée orientale.

Conclusion

Le modèle de l'Alliance HRP pour le renforcement des capacités de recherche est un modèle parmi tant d'autres. Néanmoins, il présente la caractéristique unique de permettre le développement et le renforcement des capacités de recherche des individus et des institutions grâce à l'engagement et au leadership des institutions de recherche situées dans les PRF-PRI. Ce modèle a le potentiel, en soutenant les activités pour renforcer les capacités de recherche par le biais d'institutions situées dans les régions d'intérêt, d'empêcher la fuite des cerveaux des rechercheurs qualifiés en renforçant les capacités et en offrant des opportunités viables pour la mise en œuvre de la recherche dans leur

* Brésil : Centro de Pesquisas em Saúde Reprodutiva de Campinas – CEMICAMP ; Burkina Faso : Institut de Recherche en Sciences de la Santé – IRSS ; Ghana : University of Ghana School of Public Health – UGSPH ; Kenya : African Population Health Research Center – APHRC ; Pakistan : Aga Khan University – AKU ; Thailand : Khon Kaen University – KKU ; and Viet Nam : Hanoi Medical University – HMU.

pays d'origine. Cet article sert de plan directeur à l’Alliance HRP, qui s’est fixé pour objectif d’être tenue responsable de son mandat.

Figure 1. Personnes formées dans le cadre de cours offerts par les centres ou le siège de l’Alliance HRP.

Figure 2. Étudiants en doctorat et en master qui reçoivent des bourses par le biais de l’Alliance HRP pour terminer leurs études.

Intérêts concurrents : RA, VB et AT étaient employés à l’OMS/au HRP au moment de la rédaction de ce commentaire. LB, EG, SK, PL, TTHN, SS et KT coordonnaient les centres de l’Alliance HRP dans leurs institutions et recevaient des fonds pour les gérer. Tous les auteurs déclarent ne pas avoir d’intérêts concurrents.

Financement : l’Alliance HRP est financée par le Programme spécial de recherche, de développement et de formation à la recherche en matière de reproduction humaine (HRP) des PNUD/FNUAP/UNICEF/OMS/Banque mondiale. Les points de vue de l’organisme de financement n’ont pas influencé le contenu du présent document. Cet article présente uniquement le point de vue des auteurs cités et non celui de l’OMS.

Contributions des auteurs : RA a développé les premières versions de ce commentaire avec les contributions substantielles de AT et VB. LB, EG, SK, PL, TTHN, SS et KT ont rédigé des commentaires supplémentaires sur les versions finales de ce document. Tous les auteurs ont lu et approuvé la version finale.

Remerciements : les auteurs souhaitent remercier Ian Askew pour son soutien à l’Alliance HRP et à toutes les institutions de recherche avec lesquelles ils travaillent dans le monde.

Panneau : L’Alliance HRP - vision, mission, stratégie, objectifs et valeurs fondamentales

Vision

L’Alliance HRP vise à améliorer les SDSR à l’échelle mondiale en renforçant les capacités de recherche.

Mission

Aider les institutions à développer des capacités de recherche de haute qualité en matière de SDSR.

Stratégie

En associant le renforcement des capacités de recherche à la recherche et au transfert de connaissances du HRP, l’Alliance HRP capitalise et renforce les collaborations existantes. Cela aide les institutions à se positionner sur la scène mondiale de la recherche et du transfert de connaissances en matière de SDSR. Cette aide est fournie par le biais de subventions institutionnelles à long terme aux institutions de recherche sélectionnées comme centres régionaux soutenant le renforcement des capacités de recherche des institutions de leurs régions respectives.

Objectifs

1- Renforcer la capacité de recherche en termes de SDSR dans le cadre d’une alliance d’institutions et d’intervenants dans les PFR-PRI
2- Améliorer les propres infrastructures de recherche des institutions

3- Renforcer les capacités de recherche des institutions de la région par des formations, des cours et l'éducation formelle des individus
4- Établir un lien entre la recherche du HRP et les partenaires de l'Alliance HRP sur des sujets relatifs aux SDSR
5- Mener des activités d'application des connaissances
6- Former une équipe conséquente de chercheurs de renommée mondiale dans le domaine de la recherche sur la mise en œuvre des SDSR dans le monde entier
7- Soutenir la recherche sur les questions humanitaires ou d'urgence relatives aux SDSR

Valeurs fondamentales

- Se concentrer sur l'égalité des sexes
- Promouvoir la recherche fondée sur les droits
- Mener la recherche de haut niveau sur leur mise en œuvre
- Favoriser l'application des connaissances au sein d'un réseau mondial de chercheurs en SDSR

Site Web : https://www.who.int/reproductivehealth/hrp_alliance/en/

Bibliographie

1. World Health Organization, editor. Research for universal health coverage. Geneva: WHO; 2013. 146 p. (The world health report).

2. Chu KM, Jayaraman SP, Kyamanywa P, Ntakiyiruta G. Building Research Capacity in Africa: Equity and Global Health Collaborations. PLOS Med [Internet]. 2014 Mar [cited 2019 Dec 11];11(2). Available from: https://journals.plos.org/plosmedicine/article?id=10.1371/journal.pmed.1001612

3. Belizán JM, Miller S. What can WHO do to support research in LMICs? Lancet [Internet]. 2017 Apr 29 [cited 2019 Dec 11];389. Available from: https://www.thelancet.com/journals/lancet/article/PIIS0140-6736(17)31064-4/fulltext?dgcid=recommender_referral_trendmd

4. Kabra R, Castillo M, Melián M, Ali M, Say L, Gulmezoglu AM. Research capacity strengthening for sexual and reproductive health: a case study from Latin America. Reprod Health. 2017;14:35.

5. Tulloch-Reid MK, Gore Saravia N, Dennis RJ, Jaramillo A, Cuervo LG, Walker SP, et al. Strengthening institutional capacity for equitable health research: lessons from Latin America and the Caribbean. BMJ [Internet]. 2018 [cited 2019 Dec 11];362. Available from: https://www.bmj.com/content/362/bmj.k2456

6. Bowsher G, Papamichail A, El Achi N, Ekzayez A, Roberts B, Sullivan R, et al. A narrative review of health research capacity strengthening in low and middle-income countries: lessons for conflict-affected areas. Glob Health [Internet]. 2019 [cited 2019 Dec 11];15(23). Available from: https://link.springer.com/article/10.1186/s12992-019-0465-y

7. Matus J, Walker A, Mickan S. Research capacity building frameworks for allied health professionals – a systematic review. BMC Health Serv Res [Internet]. 2018 [cited 2019 Dec 11];18(716). Available from: https://bmchealthservres.biomedcentral.com/articles/10.1186/s12913-018-3518-7

8. Kelaher M, Ng L, Knight K, Rahadi A. Equity in global health research in the new millennium: trends in first-authorship for randomized controlled trials among low- and middle-income country researchers 1990-2013. Int J Epidemiol. 2016 Dec;45(6):2174–83.

9. Hedt-Gauthier BL, Jeufack HM, Neufeld NH, Alem A, Sauer S, Odhiambo J, et al. Stuck in the middle: a systematic review of authorship in collaborative health research in Africa, 2014–2016. BMJ Glob Health. 2019 Oct;4(5):e001853.

10. Iyer AR. Authorship trends in The Lancet Global Health. Lancet Glob Health. 2018 Feb;6(2):e142.

11. WHO | HRP Alliance [Internet]. WHO. [cited 2018 Jan 6]. Available from: http://www.who.int/reproductivehealth/hrp_alliance/en/

12. Thirteenth general programme of work 2019-2023 [Internet]. [cited 2019 Dec 11]. Available from: https://www.who.int/about/what-we-do/thirteenth-general-programme-of-work-2019-2023

13. Kim CR, Tunçalp Ö, Ganatra B, Gülmezoglu AM, Group WM-AR. WHO Multi-Country Survey on Abortion-related Morbidity and Mortality in Health Facilities: study protocol. BMJ Glob Health. 2016 Nov 1;1(3):e000113.

14. Bonet M, Brizuela V, Abalos E, Cuesta C, Baguiya A, Chamillard M, et al. Frequency and management of maternal infection in health facilities in 52 countries (GLOSS): a 1-week inception cohort study. Lancet Glob Health. 2020 May 1;8(5):e661–71.

15. Tran NT, Seuc A, Coulibaly A, Landoulsi S, Millogo T, Sissoko F, et al. Post-partum family planning in Burkina Faso (Yam Daabo): a two group, multi-intervention, single-blinded, cluster-randomised controlled trial. Lancet Glob Health. 2019 Aug 1;7(8):e1109–17.

16. Bohren MA, Mehrtash H, Fawole B, Maung TM, Balde MD, Maya E, et al. How women are treated during facility-based childbirth in four countries: a cross-sectional study with labour observations and community-based surveys. The Lancet. 2019 Nov 9;394(10210):1750–63.

17. Maung TM, Show KL, Mon NO, Tunçalp Ö, Aye NS, Soe YY, et al. A qualitative study on acceptability of the mistreatment of women during childbirth in Myanmar. Reprod Health. 2020 Apr 20;17(1):56.

18. Thorson A, Aslanyan G, Brizuela V, Perez F, León RGP de, Reeder JC, et al. Research and research capacity strengthening in the context of an emerging epidemic: Zika virus in Latin America. Int J Gynecol Obstet. 2020;148(S2):1–3.
