# Supplementary material for: Strengthening research capacity through regional partners: the HRP Alliance at the World Health Organization
Source: Reprod Health. 2020 Aug 26;17:131. doi: 10.1186/s12978-020-00965-0 (PMC7448306; doi:10.1186/s12978-020-00965-0)
Supplement: Supplementary file 1 — Additional file 1. Version de l'article en français - versão do artigo em português - versión del artículo en español [file 12978_2020_965_MOESM1_ESM.zip › HRP RCS commentary_PO.pdf]

Reforçando a capacidade de pesquisa por meio de parceiros regionais: a HRP Alliance na Organização Mundial da Saúde

Resumo

Introducção: As melhorias na saúde não podem ocorrer sem as pesquisas de ponta que informam o planejamento e a implementação de programas e políticas de saúde, evidenciando a necessidade de pesquisadores e instituições capazes e qualificadas em países onde a incidência de doença é alta, e os recursos são limitados.

Texto principal: O empenho pelo reforço da capacidade de pesquisa (RCS) em países de baixa e média renda (LMIC) tem incluído provisões de bolsas de formação para pós-graduação, geralmente em países de renda alta, estágios em universidades/centros de pesquisas, cursos de curta duração, assim como envolvimento com grupos de pesquisas para experiência prática, entre outros. A HRP Alliance fornece oportunidades para o desenvolvimento de capacidade de pesquisa local em saúde e direitos sexuais e reprodutivos (SRHR) através de instituições estabelecidas em países de baixa e média renda (LMIC) ligados a estudos colaborativos anteriores e em andamento. É uma rede de instituições parceiras HRP, escritórios nacionais e regionais da OMS, programas e parcerias especiais e centros colaborativos da OMS.

Conclusão: É através da HRP Alliance que o desenvolvimento e treinamento em pesquisa em reprodução humana (HRP) busca melhorar a saúde da população pelo reforço local na capacidade de pesquisa em SRHR em todo o mundo, com foco nos LMIC, em alinhamento com a busca da OMS em promover populações mais saudáveis.

Palabras chave: reforço da capacidade de pesquisa, pesquisa, saúde sexual e reprodutiva, países de baixa e média renda

Introducção

As melhorias na saúde não podem ocorrer sem as pesquisas de ponta que informam o planejamento e a implementação de programas e políticas de saúde. As evidências geradas no país são necessárias para moldar e adicionar relevância às agendas nacionais de pesquisas e políticas.(1–3) Isso destaca a necessidade por pesquisas e instituições qualificadas e capazes em países onde a incidência de doenças é alta e os recursos são limitados. A inversa relação entre a incidência de doenças relacionadas a saúde e direitos sexuais e reprodutivos (SRHR) e a capacidade de pesquisa disponível é conhecida e diferentes esforços têm sido tomados para o reforço na capacidade de países de baixa e média renda (LMIC) nas últimas décadas. (4)

Texto principal

O empenho pelo reforço da capacidade de pesquisa (RCS) em países de baixa e média renda (LMIC) tem incluído provisões de bolsas de formação para pós-graduação, geralmente em países de alta renda, estágios em universidades/centros de pesquisas, cursos de curta duração, assim como envolvimento com grupos de pesquisas para experiência prática, entre outros. (5–7) Esses esforços têm resultado na formação de pesquisadores para países de baixa e média renda (LMIC), mas nem sempre aumentaram a capacidade de instituições de LMICs a conduzirem as próprias pesquisas para identificar ou monitorar o conhecimento e o uso de melhores resultados de SRHR.(6) O RCS precisa desenvolver a capacidade das instituições-alvo nos países em desenvolvimento, para que possam treinar com sucesso pesquisadores competentes e responder às agendas locais, regionais e globais.(5,6) Enquanto existem atualmente institutos bem-estabelecidos de pesquisa em países de baixa e média renda (LMIC) que possam treinar e conduzir pesquisas de alta qualidade, ainda existem falhas no que diz respeito ao reforço da capacidade de pesquisa de pesquisadores júnior. Garantir autoria justa e igualitária também é fundamental para o desenvolvimento local do reforço da capacidade de pesquisa (RCS).(8–10)

A HRP Alliance

A HRP Alliance, criada em 2016 como parte do PNUD/UNFPA/UNICEF/OMS/World Bank Special Programme of Research, Desenvolvimento e Treinamento em Pesquisa em Reprodução Humana (HRP), (11) fornece oportunidades para o desenvolvimento da capacidade de pesquisa local através de instituições ligadas a estudos colaborativos anteriores e em andamento (visite o site pelo link). É uma rede de instituições parceiras HRP, escritórios nacionais e regionais da OMS, programas e parcerias especiais e centros colaborativos da OMS. É através da HRP Alliance que o HRP busca melhorar a saúde da população pelo reforço local na capacidade de pesquisa em SRHR em todo o mundo, com foco nos países de baixa e média renda (LMIC), em alinhamento com a busca da OMS em promover populações mais saudáveis. (12) (Ver Painel). Apesar da recente criação da HRP Alliance, a HRP lidera os esforços da RCS há várias décadas. No passado, isso era feito por meio de subsídios de desenvolvimento institucional de longo prazo, focados principalmente no fortalecimento institucional individual e no apoio a projetos de pesquisa locais. O foco agora, através da HRP Alliance, reside na construção de uma massa crítica regional de pesquisadores apoiados por instituições localizadas nas regiões, ilustrando uma verdadeira colaboração horizontal entre pesquisadores.

No cerne da HRP Alliance estão os “centros” de reforços da capacidade de pesquisa (RCS), selecionados através de um processo competitivo acessível que leva em consideração a experiência

em saúde e direitos sexuais e reprodutivos, e a capacidade para fornecer liderança regional em RCS. Estes centros, estabelecidos no Brasil, Burkina Faso, Gana, Quênia, Paquistão, Tailândia e Vietnã,[*] são encarregados de fornecer suporte de RCS às instituições em suas regiões. Enquanto a HRP Alliance ainda está em seus estágios iniciais, o suporte é fornecido principalmente através de:

- Workshops e treinamentos sobre saúde e direitos sexuais e reprodutivos (SRHR), metodologias de pesquisa e bioestatística, revisão sistemática e metanálise, métodos de pesquisa qualitativa, implementação de pesquisa, monitoramento e avaliação, desenvolvimento de protocolo e redação (Figura 1);
- Cursos de pós-graduação específicos para pesquisas em SRHR (através de cursos de mestrado e doutorado) (Figura 2);
- Suporte personalizado para institutos de pesquisa nacionais no desenvolvimento e implementação de pesquisas e produção de publicações científicas;
- Liderança em atividades de transferência de conhecimento que contribuem para garantir a implementação das recomendações da OMS para políticas e práticas;
- Propostas de subsídios colaborativas entre vários centros ou instituições apoiadas pelos centros usando a rede HRP Alliance para alavancar experiência e conhecimento;
- Colaborações permitidas entre os bolsistas da HRP Alliance para projetos específicos de pesquisa;
- Resposta a emergências de saúde por meio da pesquisa SRHR para melhorar a resposta rápida do sistema de saúde e a capacidade local de pesquisa.

Desde o início, a HRP Alliance treinou mais de 700 participantes dos LMIC em 30 oficinas e cursos e apoia mais de 60 pesquisadores na obtenção de mestrado ou doutorado, alguns dos quais envolvidos na implementação local de estudos(13–17) multinacionais do HRP e em estudos secundários de análises. A HRP Alliance apoia o uso de autoria de grupo para estudos em vários países, liderança local em análises secundárias e específicas de cada país, e o estabelecimento de regras e funções de autoria antes do início do projeto. Mais de 20 grupos de pesquisa da América Latina foram financiados pela HRP Alliance com o objetivo de fornecer base de evidências em resposta à epidemia do vírus Zica em 2016-2017 (18) e à crise migratória em massa nas Américas em 2019-2020. A HRP Alliance também responde rapidamente às decorrentes necessidades de emergências humanitárias e de saúde, de acordo com as demandas específicas de RCS e pesquisa. Nos próximos anos, a HRP Alliance apoiará pesquisadores júnior por meio de um programa de orientação personalizado para mulheres e bolsas de pós-doutorado, além de apoiar pesquisas adicionais para estudar o SRHR de migrantes na região do Mediterrâneo Oriental.

Conclusão

O modelo HRP Alliance para RCS é um dentre muitos, mas possui a característica única de permitir o desenvolvimento e o aumento da capacidade de pesquisa de indivíduos e instituições por meio do engajamento e liderança de instituições de pesquisa localizadas em países de baixa e média renda (LMIC). Esse modelo tem o potencial, apoiando as atividades de RCS por meio de instituições localizadas nas regiões de interesse, para impedir a futura fuga de cérebros de pesquisadores qualificados, fortalecendo a capacidade e oferecendo oportunidades viáveis para a implementação

[*] Brasil: Centro de Pesquisas em Saúde Reprodutiva de Campinas – CEMICAMP; Burkina Faso: Institut de Recherche en Sciences de la Santé – IRSS; Ghana: University of Ghana School of Public Health – UGSPH; Kenya: African Population Health Research Center – APHRC; Pakistan: Aga Khan University – AKU; Thailand: Khon Kaen University – KKU; and Viet Nam: Hanoi Medical University – HMU.

de pesquisas em seus países de origem. Este artigo serve como um modelo do que a HRP Alliance se propôs a fazer e do que se responsabiliza em seu mandato.

Figura 1. Indivíduos treinados através de cursos oferecidos pelos centros HRP Alliance ou pela sede HRP Alliance.

Figura 2. Estudantes de mestrado e doutorado recebendo bolsas de estudos através da HRP Alliance para concluir seus estudos.

Conflitos de interesses: RA, VB e AT foram empregados na OMS / HRP no momento da produção deste comentário. LB, EG, SK, PL, TTHN, SS e KT estavam coordenando os centros da HRP Alliance em suas instituições e recebendo fundos para gerenciá-los. Todos os autores declaram não ter conflitos de interesses.

Financiamento: a HRP Alliance é financiada pela PNUD/UNFPA/UNICEF/OMS/World Bank Special Programme of Research, Desenvolvimento e Treinamento em Pesquisa em Reprodução Humana (HRP). As opiniões do órgão financiador não influenciaram o conteúdo deste documento. Este artigo representa os pontos de vista dos autores nomeados e não representam as opiniões da OMS.

Contribuições dos autores: RA desenvolveu as primeiras versões deste comentário com contribuições substanciais de AT e VB. LB, EG, SK, PL, TTHN, SS e KT forneceram comentários adicionais sobre as versões finais deste documento. Todos os autores leram e aprovaram a versão final.

Agradecimentos: os autores desejam agradecer a Ian Askew por seu apoio à HRP Alliance e a todas as instituições de pesquisa com quem trabalham globalmente.

**Painel: A HRP Alliance - visão, missão, estratégia, objetivos e valores fundamentais**
**Visão**<br>A HRP Alliance visa melhorar o SRHR globalmente, reforçando a capacidade de pesquisa.<br>**Missão**<br>Apoiar as instituições a desenvolver capacidade de pesquisa de alta qualidade em SRHR.<br>**Estratégia**<br>Ao vincular o reforço da capacidade de pesquisa (RCS) à pesquisa e à transferência de conhecimento em HRP, a HRP Alliance investe e reforça as colaborações existentes. A HRP Alliance fornece apoio para instituições para que se posicionem na arena global de pesquisa e transferência de conhecimento em SRHR. Isso é proporcionado por meio de subsídios institucionais de longo prazo para instituições de pesquisa selecionadas como centros regionais de apoio ao RCS para instituições em suas respectivas regiões.<br>**Objetivos**<br>1- Fortalecer a capacidade de pesquisa de SRHR através de uma aliança entre instituições e partes interessadas nos países de baixa e média renda (LMIC)<br>2- Para melhorar a infraestrutura de pesquisa das próprias instituições

3- Para reforçar a capacidade de pesquisa das instituições nas regiões através de treinamentos, cursos, e formação educacional de indivíduos
4- Vincular a pesquisa de HRP com os parceiros da HRP Alliance sobre tópicos de SRHR
5- Liderar atividades de translação de conhecimento
6- Construir uma massa crítica de pesquisadores de classe mundial na pesquisa de implementação de SRHR em todo o mundo
7- Apoiar a pesquisa em questões humanitárias ou emergenciais de SRHR

Valores fundamentais

- Focar em igualdade de gêneros
- Promover pesquisa baseada em direitos
- Liderar a implementação de pesquisas de alto nível
- Fomentar a transferência de conhecimento entre uma rede global de pesquisadores da SRHR

Site: https://www.who.int/reproductivehealth/hrp_alliance/en/

Bibliografia

1. World Health Organization, editor. Research for universal health coverage. Geneva: WHO; 2013. 146 p. (The world health report).

2. Chu KM, Jayaraman SP, Kyamanywa P, Ntakiyiruta G. Building Research Capacity in Africa: Equity and Global Health Collaborations. PLOS Med [Internet]. 2014 Mar [cited 2019 Dec 11];11(2). Available from: https://journals.plos.org/plosmedicine/article?id=10.1371/journal.pmed.1001612

3. Belizán JM, Miller S. What can WHO do to support research in LMICs? Lancet [Internet]. 2017 Apr 29 [cited 2019 Dec 11];389. Available from: https://www.thelancet.com/journals/lancet/article/PIIS0140-6736(17)31064-4/fulltext?dgcid=recommender_referral_trendmd

4. Kabra R, Castillo M, Melián M, Ali M, Say L, Gulmezoglu AM. Research capacity strengthening for sexual and reproductive health: a case study from Latin America. Reprod Health. 2017;14:35.

5. Tulloch-Reid MK, Gore Saravia N, Dennis RJ, Jaramillo A, Cuervo LG, Walker SP, et al. Strengthening institutional capacity for equitable health research: lessons from Latin America and the Caribbean. BMJ [Internet]. 2018 [cited 2019 Dec 11];362. Available from: https://www.bmj.com/content/362/bmj.k2456

6. Bowsher G, Papamichail A, El Achi N, Ekzayez A, Roberts B, Sullivan R, et al. A narrative review of health research capacity strengthening in low and middle-income countries: lessons for conflict-affected areas. Glob Health [Internet]. 2019 [cited 2019 Dec 11];15(23). Available from: https://link.springer.com/article/10.1186/s12992-019-0465-y

7. Matus J, Walker A, Mickan S. Research capacity building frameworks for allied health professionals – a systematic review. BMC Health Serv Res [Internet]. 2018 [cited 2019 Dec 11];18(716). Available from: https://bmchealthservres.biomedcentral.com/articles/10.1186/s12913-018-3518-7

8. Kelaher M, Ng L, Knight K, Rahadi A. Equity in global health research in the new millennium: trends in first-authorship for randomized controlled trials among low- and middle-income country researchers 1990-2013. Int J Epidemiol. 2016 Dec;45(6):2174–83.

9. Hedt-Gauthier BL, Jeufack HM, Neufeld NH, Alem A, Sauer S, Odhiambo J, et al. Stuck in the middle: a systematic review of authorship in collaborative health research in Africa, 2014–2016. BMJ Glob Health. 2019 Oct;4(5):e001853.

10. Iyer AR. Authorship trends in The Lancet Global Health. Lancet Glob Health. 2018 Feb;6(2):e142.

11. WHO | HRP Alliance [Internet]. WHO. [cited 2018 Jan 6]. Available from: http://www.who.int/reproductivehealth/hrp_alliance/en/

12. Thirteenth general programme of work 2019-2023 [Internet]. [cited 2019 Dec 11]. Available from: https://www.who.int/about/what-we-do/thirteenth-general-programme-of-work-2019-2023

13. Kim CR, Tunçalp Ö, Ganatra B, Gülmezoglu AM, Group WM-AR. WHO Multi-Country Survey on Abortion-related Morbidity and Mortality in Health Facilities: study protocol. BMJ Glob Health. 2016 Nov 1;1(3):e000113.

14. Bonet M, Brizuela V, Abalos E, Cuesta C, Baguiya A, Chamillard M, et al. Frequency and management of maternal infection in health facilities in 52 countries (GLOSS): a 1-week inception cohort study. Lancet Glob Health. 2020 May 1;8(5):e661–71.

15. Tran NT, Seuc A, Coulibaly A, Landoulsi S, Millogo T, Sissoko F, et al. Post-partum family planning in Burkina Faso (Yam Daabo): a two group, multi-intervention, single-blinded, cluster-randomised controlled trial. Lancet Glob Health. 2019 Aug 1;7(8):e1109–17.

16. Bohren MA, Mehrtash H, Fawole B, Maung TM, Balde MD, Maya E, et al. How women are treated during facility-based childbirth in four countries: a cross-sectional study with labour observations and community-based surveys. The Lancet. 2019 Nov 9;394(10210):1750–63.

17. Maung TM, Show KL, Mon NO, Tunçalp Ö, Aye NS, Soe YY, et al. A qualitative study on acceptability of the mistreatment of women during childbirth in Myanmar. Reprod Health. 2020 Apr 20;17(1):56.

18. Thorson A, Aslanyan G, Brizuela V, Perez F, León RGP de, Reeder JC, et al. Research and research capacity strengthening in the context of an emerging epidemic: Zika virus in Latin America. Int J Gynecol Obstet. 2020;148(S2):1–3.
