# Supplementary material for: Strengthening research capacity through regional partners: the HRP Alliance at the World Health Organization
Source: Reprod Health. 2020 Aug 26;17:131. doi: 10.1186/s12978-020-00965-0 (PMC7448306; doi:10.1186/s12978-020-00965-0)
Supplement: Supplementary file 1 — Additional file 1. Version de l'article en français - versão do artigo em português - versión del artículo en español [file 12978_2020_965_MOESM1_ESM.zip › HRP RCS commentary_SP.pdf]

Fortaleciendo la capacidad de investigación mediante contrapartes regionales: la Alianza HRP en la Organización Mundial de la Salud

Resumen

Introducción: Las mejoras en la salud no pueden suceder sin investigaciones de vanguardia que informen sobre el diseño y la implementación de programas y políticas de salud y que pongan énfasis en la necesidad de contar con investigadores e instituciones capaces y competentes en los países donde la carga de morbilidad es alta y los recursos son limitados.

Texto principal: Los esfuerzos para el fortalecimiento de la capacidad de investigación (RCS) en los países de ingresos bajos y medianos incluyeron, entre otros, el ofrecimiento de becas de capacitación a graduados universitarios, a menudo en países de ingresos altos, pasantías en universidades y centros de investigación, cursos breves y también participación en grupos de investigación para experiencias prácticas. La Alianza HRP ofrece oportunidades para el desarrollo de capacidades de investigación locales en salud y derechos sexuales y reproductivos (SDSR) a través de instituciones ubicadas en países de ingresos bajos y medianos vinculadas con estudios colaborativos presentes y pasados. Se trata de una red de instituciones asociadas de investigación del Programa de Reproducción Humana (HRP), oficinas regionales y nacionales de la OMS, programas y asociaciones especiales de la OMS y centros colaboradores de la OMS.

Conclusión: A través de la Alianza HRP, el HRP busca mejorar la salud de la población con el fortalecimiento de la capacidad de investigación local en SDSR en todo el mundo, centrado en los países de ingresos bajos y medianos, y alineado con la misión de la OMS de promover poblaciones más saludables.

Palabras clave: fortalecimiento de la capacidad de Investigación, investigación, salud sexual y reproductiva, países de ingresos bajos y medianos

Introducción

Las mejoras en la salud no pueden suceder sin investigaciones de vanguardia que informen sobre el diseño y la implementación de programas y políticas de salud. La evidencia generada dentro de los países es necesaria para dar forma y mayor relevancia a las investigaciones y las agendas políticas nacionales.(1–3) Esto resalta la necesidad de contar con investigadores e instituciones capaces y competentes en los países donde la carga de morbilidad es alta y los recursos son limitados. Se reconoce la relación inversa entre la carga de morbilidad relacionada con la salud y los derechos sexuales y reproductivos (SDSR) y la disponibilidad de la capacidad de investigación; y se han llevado a cabo distintos esfuerzos para fortalecer la capacidad de los países de ingresos bajos y medianos en las últimas décadas.(4)

Texto principal

Los esfuerzos para el fortalecimiento de la capacidad de investigación (RCS) en los países de ingresos bajos y medianos incluyeron, entre otros, el ofrecimiento de becas de capacitación a graduados universitarios, a menudo en países de ingresos altos, pasantías en universidades y centros de investigación, cursos breves y también participación en grupos de investigación para experiencias prácticas.(5–7) Gracias a estos esfuerzos, se han producido investigadores en los países de ingresos bajos y medianos, pero no siempre se logró desarrollar la capacidad de las instituciones de estos países para realizar sus propias investigaciones con el objetivo de identificar soluciones o monitorear el uso y la adopción de mejores resultados de SDSR.[6] Para que el RCS sea sustentable, debe desarrollar la capacidad de las instituciones destinatarias en los países que la necesitan de modo que puedan capacitar adecuadamente a los investigadores competentes y responder a las agendas locales, regionales y globales.(5,6) Si bien, en la actualidad, existen institutos de investigación consolidados en los países de ingresos bajos y medianos que pueden brindar capacitación y realizar investigaciones de buena calidad, aún existen carencias con respecto al fortalecimiento de la capacidad de investigación de los investigadores júnior. Garantizar la autoría equitativa y justa también es fundamental para el desarrollo del RCS.(8–10)

La Alianza HRP
La Alianza HRP, creada en 2016 como parte del Programa Especial del PNUD/UNFPA/UNICEF/OMS/Banco Mundial de Investigaciones, Desarrollo y Formación de Investigadores sobre Reproducción Humana (HRP),(11) ofrece oportunidades para el desarrollo de la capacidad de investigación local a través de instituciones vinculadas con estudios colaborativos presentes y pasados (visite el sitio web desde este enlace). Se trata de una red de instituciones asociadas de investigación del HRP, oficinas regionales y nacionales de la OMS, programas y asociaciones especiales de la OMS y centros colaboradores de la OMS. A través de la Alianza HRP, el HRP busca mejorar la salud de la población con el fortalecimiento de la capacidad de investigación local en SDSR en todo el mundo, centrado en los países de ingresos bajos y medianos, y alineado con la misión de la OMS de promover poblaciones más saludables.(12) (Ver el Panel). A pesar de la reciente creación de la Alianza HRP, HRP ha estado liderando esfuerzos en RCS por décadas. En el pasado, esto se realizaba mediante fondos de desarrollo institucional de largo plazo que se enfocaban principalmente en fortalecimiento institucional individual y el apoyo de proyectos de investigación locales. El foco ahora, a través de la Alianza HRP, yace en crear una masa crítica regional de investigadores apoyados por instituciones ubicadas en las regiones, ilustrando así una verdadera colaboración horizontal entre investigadores.

El núcleo de la Alianza HRP son los “nodos” de RCS regionales, seleccionados mediante un proceso competitivo abierto que considera la experiencia en el RCS en SDSR, y la capacidad de liderazgo regional en RCS. Estos nodos, con base en Brasil, Burkina Faso, Ghana, Kenia, Pakistán, Tailandia y

Vietnam,* están encargados de brindar respaldo para el RCS a las instituciones de sus regiones. Si bien la Alianza HRP aún se encuentra en la etapa inicial, recibe respaldo principalmente de la siguiente manera:

- Talleres y capacitaciones sobre SDSR, metodologías de investigación y bioestadística, revisión sistemática y metanálisis, métodos de investigación cualitativa, investigación de la implementación, monitoreo y evaluación, desarrollo de protocolos y redacción de manuscritos (Figura 1)
- Educación de posgrado específica sobre investigación en SDSR (mediante maestrías o doctorados) (Figura 2)
- Respaldo individualizado a las instituciones de investigación de cada país para el desarrollo y la implementación de estudios de investigación y la producción de publicaciones científicas.
- Liderazgo en actividades de transferencia de conocimientos para garantizar la implementación de las recomendaciones de la OMS relativas a políticas y prácticas
- Propuestas de becas colaborativas entre varios nodos o instituciones apoyadas por los nodos usando la red de la Alianza HRP para aprovechar la experiencia
- Posibilidades de colaboraciones entre los becarios de la Alianza HRP para proyectos de investigación específicos
- Respuesta a emergencias sanitarias mediante investigaciones en SDSR para mejorar la rápida respuesta del sistema de salud y la capacidad de investigación local

Desde el comienzo, la Alianza HRP ha capacitado a más de 700 participantes de países de ingresos bajos y medianos a través de 30 talleres y cursos y respalda a más de 60 investigadores para que obtengan sus maestrías o doctorados, algunos de los cuales han participado en la implementación local de los estudios multinacionales del HRP(13–17) y en los análisis secundarios. La Alianza HRP respalda el uso de la autoría grupal para los estudios multinacionales, el liderazgo local en los análisis secundarios y específicos de cada país y el establecimiento de normas y funciones relacionadas con la autoría antes de la puesta en marcha del proyecto. Más de 20 grupos de investigación de América Latina han recibido financiación de la Alianza HRP para generar la base de la evidencia en respuesta a la epidemia del virus del Zika en 2016-2017 (18) y a la crisis de migración masiva en América en 2019-2020. Asimismo, la Alianza HRP responde rápidamente a las necesidades que surgen a partir de las emergencias sanitarias y humanitarias según las necesidades específicas para el RCS y la investigación. En los próximos años, la Alianza HRP respaldará a los investigadores júnior con un programa de mentoría individualizado para mujeres y becas posdoctorales, además de investigaciones adicionales para estudiar la SDSR de los migrantes en la región del Mediterráneo Oriental.

Conclusión

El modelo de la Alianza HRP para el RCS es uno de muchos, pero tiene una característica exclusiva y es que permite el desarrollo y la construcción de la capacidad de investigación de las personas y las instituciones mediante el compromiso y el liderazgo de las instituciones de investigación ubicadas en países de ingresos bajos y medianos. Este modelo tiene el potencial, mediante el apoyo de actividades de RCS por instituciones ubicadas en las regiones de interés, de prevenir la fuga de cerebros de investigadores calificados al fortalecer la capacidad y ofrecer oportunidades viables para implementar estudios de investigación en sus propios países. Este artículo sirve como anteproyecto de lo que la Alianza HRP se ha propuesto llevar a cabo y sus responsabilidades conforme a su mandato.

---

* Brasil: Centro de Pesquisas em Saúde Reprodutiva de Campinas (CEMICAMP); Burkina Faso: Institut de Recherche en Sciences de la Santé (IRSS); Ghana: University of Ghana School of Public Health (UGSPH); Kenia: African Population Health Research Center (APHRC); Pakistán: Aga Khan University (AKU); Tailandia: Khon Kaen University (KKU); y Vietnam: Hanoi Medical University (HMU).

Figura 1. Individuos capacitados mediante cursos ofrecidos por los nodos de la Alianza HRP o por la Alianza HRP en la sede central.

Figura 2. Estudiantes de maestrías y doctorados que reciben becas otorgadas por la Alianza HRP para completar sus estudios.

Conflicto de intereses: RA, VB y AT eran empleados de la OMS/HRP al momento de la redacción de este comentario. LB, EG, SK, PL, TTHN, SS y KT coordinaban los nodos de la Alianza HRP en sus instituciones y recibían fondos para su gestión. Todos los autores declaran que no tienen conflicto de intereses.

Financiación: la Alianza HRP está financiada por el Programa Especial del PNUD/UNFPA/UNICEF/OMS/Banco Mundial de Investigaciones, Desarrollo y Formación de Investigadores sobre Reproducción Humana (HRP). Las opiniones del organismo financiador no afectaron el contenido de este manuscrito. Este artículo representa solamente las opiniones de los autores mencionados y no las de la OMS.

Contribuciones de los autores: RA desarrolló las primeras versiones de este comentario, con la ayuda esencial de AT y VB. LB, EG, SK, PL, TTHN, SS y KT brindaron sus opiniones adicionales sobre las versiones finales de este manuscrito. Todos los autores leyeron y aprobaron la versión final.

Agradecimientos: los autores desean agradecer a Ian Askew por su respaldo de la Alianza HRP y a todas las instituciones de investigación con las que trabajan globalmente.

***Panel*: Alianza HRP: visión, misión, estrategia, metas y valores fundamentales**

Visión

El objetivo de la Alianza HRP es mejorar la SDSR a nivel global mediante el fortalecimiento de la capacidad de investigación.

Misión

Respaldar a las instituciones para que desarrollen su capacidad de investigación en SDSR de buena calidad.

Estrategia

Mediante la vinculación del fortalecimiento de la capacidad de investigación (RCS) con la investigación y la transferencia de conocimientos del HRP, la Alianza HRP capitaliza y refuerza las colaboraciones existentes. Brinda respaldo a las instituciones para que logren posicionarse en la investigación sobre SDSR global y en el campo de la transferencia de conocimientos. Este se ofrece mediante subsidios institucionales a largo plazo otorgados a instituciones de investigación seleccionadas como nodos regionales de RCS en sus regiones respectivas.

Metas

1- Fortalecer la capacidad de investigación en SDSR en una alianza de instituciones y partes interesadas en países de ingresos bajos y medianos.
2- Mejorar la propia infraestructura de investigación de las instituciones.
3- Fortalecer la capacidad de investigación de las instituciones en la región mediante capacitaciones, cursos y educación formal para individuos.

4- Vincular la investigación del HRP con los asociados de la Alianza HRP sobre temas de SDSR.
5- Dirigir las actividades de traducción de conocimientos.
6- Construir una masa crítica de investigadores de primera categoría en investigación de la implementación de SDSR en todo el mundo.
7- Respaldar la investigación sobre cuestiones de SDSR humanitarios o de emergencia.

Valores fundamentales

- Centrarse en la igualdad de género.
- Fomentar la investigación basada en los derechos.
- Dirigir la investigación de la implementación de buena calidad.
- Promover la traducción de conocimientos en una red global de investigadores de SDSR.

Sitio web: https://www.who.int/reproductivehealth/hrp_alliance/en/

Bibliografía

1. World Health Organization, editor. Research for universal health coverage. Geneva: WHO; 2013. 146 p. (The world health report).

2. Chu KM, Jayaraman SP, Kyamanywa P, Ntakiyiruta G. Building Research Capacity in Africa: Equity and Global Health Collaborations. PLOS Med [Internet]. 2014 Mar [cited 2019 Dec 11];11(2). Available from: https://journals.plos.org/plosmedicine/article?id=10.1371/journal.pmed.1001612

3. Belizán JM, Miller S. What can WHO do to support research in LMICs? Lancet [Internet]. 2017 Apr 29 [cited 2019 Dec 11];389. Available from: https://www.thelancet.com/journals/lancet/article/PIIS0140-6736(17)31064-4/fulltext?dgcid=recommender_referral_trendmd

4. Kabra R, Castillo M, Melián M, Ali M, Say L, Gulmezoglu AM. Research capacity strengthening for sexual and reproductive health: a case study from Latin America. Reprod Health. 2017;14:35.

5. Tulloch-Reid MK, Gore Saravia N, Dennis RJ, Jaramillo A, Cuervo LG, Walker SP, et al. Strengthening institutional capacity for equitable health research: lessons from Latin America and the Caribbean. BMJ [Internet]. 2018 [cited 2019 Dec 11];362. Available from: https://www.bmj.com/content/362/bmj.k2456

6. Bowsher G, Papamichail A, El Achi N, Ekzayez A, Roberts B, Sullivan R, et al. A narrative review of health research capacity strengthening in low and middle-income countries: lessons for conflict-affected areas. Glob Health [Internet]. 2019 [cited 2019 Dec 11];15(23). Available from: https://link.springer.com/article/10.1186/s12992-019-0465-y

7. Matus J, Walker A, Mickan S. Research capacity building frameworks for allied health professionals – a systematic review. BMC Health Serv Res [Internet]. 2018 [cited 2019 Dec 11];18(716). Available from: https://bmchealthservres.biomedcentral.com/articles/10.1186/s12913-018-3518-7

8. Kelaher M, Ng L, Knight K, Rahadi A. Equity in global health research in the new millennium: trends in first-authorship for randomized controlled trials among low- and middle-income country researchers 1990-2013. Int J Epidemiol. 2016 Dec;45(6):2174–83.

9. Hedt-Gauthier BL, Jeufack HM, Neufeld NH, Alem A, Sauer S, Odhiambo J, et al. Stuck in the middle: a systematic review of authorship in collaborative health research in Africa, 2014–2016. BMJ Glob Health. 2019 Oct;4(5):e001853.

10. Iyer AR. Authorship trends in The Lancet Global Health. Lancet Glob Health. 2018 Feb;6(2):e142.

11. WHO | HRP Alliance [Internet]. WHO. [cited 2018 Jan 6]. Available from: http://www.who.int/reproductivehealth/hrp_alliance/en/

12. Thirteenth general programme of work 2019-2023 [Internet]. [cited 2019 Dec 11]. Available from: https://www.who.int/about/what-we-do/thirteenth-general-programme-of-work-2019-2023

13. Kim CR, Tunçalp Ö, Ganatra B, Gülmezoglu AM, Group WM-AR. WHO Multi-Country Survey on Abortion-related Morbidity and Mortality in Health Facilities: study protocol. BMJ Glob Health. 2016 Nov 1;1(3):e000113.

14. Bonet M, Brizuela V, Abalos E, Cuesta C, Baguiya A, Chamillard M, et al. Frequency and management of maternal infection in health facilities in 52 countries (GLOSS): a 1-week inception cohort study. Lancet Glob Health. 2020 May 1;8(5):e661–71.

15. Tran NT, Seuc A, Coulibaly A, Landoulsi S, Millogo T, Sissoko F, et al. Post-partum family planning in Burkina Faso (Yam Daabo): a two group, multi-intervention, single-blinded, cluster-randomised controlled trial. Lancet Glob Health. 2019 Aug 1;7(8):e1109–17.

16. Bohren MA, Mehrtash H, Fawole B, Maung TM, Balde MD, Maya E, et al. How women are treated during facility-based childbirth in four countries: a cross-sectional study with labour observations and community-based surveys. The Lancet. 2019 Nov 9;394(10210):1750–63.

17. Maung TM, Show KL, Mon NO, Tunçalp Ö, Aye NS, Soe YY, et al. A qualitative study on acceptability of the mistreatment of women during childbirth in Myanmar. Reprod Health. 2020 Apr 20;17(1):56.

18. Thorson A, Aslanyan G, Brizuela V, Perez F, León RGP de, Reeder JC, et al. Research and research capacity strengthening in the context of an emerging epidemic: Zika virus in Latin America. Int J Gynecol Obstet. 2020;148(S2):1–3.
